# Peter Kwong Discusses Structural Biology, Antibody-Mediated Immunity, and Vaccine Design

**DOI:** 10.20411/pai.v11i2.1129

**Published:** 2026-11-02

**Authors:** Neil S. Greenspan

**Affiliations:** 1 Case Western Reserve University, Cleveland, Ohio

**Keywords:** HIV-1, Antibody Ontogeny, Broadly Neutralizing Antibodies, Structure-based Vaccine Design, Structural Biology

## Abstract

Peter D. Kwong, PhD, is an internationally recognized structural biologist and director of the Aaron Diamond AIDS Research Center at Columbia University. In this interview with Neil S. Greenspan, senior editor of *Pathogens and Immunity*, Dr. Kwong discusses his work in advancing the understanding of biological macromolecules, especially proteins, and how it has helped connect structural biology with antibody-mediated immunity and vaccine design. His contributions have earned numerous honors, including the Burroughs Wellcome Fund Career Award, the Presidential Early Career Award, NIH Director’s Awards, NIAID Merit Awards, and the Norman P. Salzman Award in Virology.

## NEIL S. GREENSPAN, MD, PHD

I am Neil Greenspan, a senior editor at *Pathogens and Immunity*, which is a research journal based at Case Western Reserve University. Today I’m delighted to have with me Peter Kwong, PhD, who is currently the Richard J. Stock Professor of Medical Sciences of Infectious Diseases in Medicine and Professor of Biochemistry and Molecular Biophysics at Columbia University in New York City.

Peter is an internationally known structural biologist and the Director of the Aaron Diamond AIDS Research Center (ADARC). After completing his undergraduate studies in chemistry and physics, as well as a master’s degree in biochemistry and molecular biology at the University of Chicago, Peter went to Columbia for graduate and postdoctoral work with Wayne Hendrickson, the eminent structural biologist himself. He then founded and directed the Structural Biology Section at the Vaccine Research Center (VRC) of the National Institutes of Health (NIH) where he was Section Chief for 23 years.

Peter returned to New York City and Columbia in 2023. As you will hear in our conversation, Peter has been a major force in bringing insights from structural studies of biological macromolecules, especially proteins, to the study of antibody-mediated immunity and vaccine design. He has been the recipient of numerous honors and awards, including, but not limited to, the 1998 Burroughs Wellcome Fund Career Award in Biomedical Sciences, the 2003 Presidential Early Career Award for Scientists and Engineers, both of which are very prestigious.

He’s a fellow of the American Society for Microbiology. In 2007, he received the Director’s Award of the National Institutes of Health, which is also quite notable, multiple NIAID Merit Awards (National Institute of Allergy and Infectious Diseases), and the Norman P. Salzman Award in Virology. And there are others, and I would be willing to wager, perhaps not on a betting market, that he’ll be getting future awards.

Welcome Peter. Can you summarize your educational background and scientific training if you have anything to add to what I’ve already articulated?

## PETER D. KWONG, PHD

Thank you for the wonderful introduction. I’m delighted to speak with you about how the work that I’ve done relates to *Pathogens and Immunity* and specifically how we can potentially make structure-based vaccines. The only thing that’s a little bit different is that I started with Paul Sigler at the University of Chicago, as you mentioned, but when I went to Wayne Hendrickson, I not only received my PhD there in 1995, but I actually stayed on to 2000, partly because I got little crystals of gp120 and felt I had to do the structure [1, 2, 3]. After that was on the covers of *Nature* and *Science* in 1998, I figured I could get a job. Eventually I got a job, and as you said, went to the NIH in 2000 to found the Structural Biology Section at the VRC, NIH.

One other footnote was that, when I went to the NIH, Washington, D.C. wasn’t that far away from New York City, and I love New York. And starting about 2009, 2010, I started a collaboration with Larry Shapiro at Columbia University on Antibodyomics [4] and used to spend about 5 or 10 days a month going back and forth, looking at antibodies, informatics systems, and other things. I became an Adjunct Professor in Columbia University’s Department of Biochemistry in 2016. Eventually, I accepted a job from David Ho, to take over from him as the next ADARC Director in 2019, but because of the pandemic in 2020, I delayed my start until December 2023. So, it’s only been about 2.5 years that I’ve been formally here at Columbia.

## NSG

You had an affiliation in the intervening period?

## PDK

That’s correct.

## NSG

Are there any particular experiences or people — you mentioned Paul Sigler briefly — that were especially significant in guiding you to a research career and to structural biology in particular?

## PDK

I mentioned Paul Sigler earlier because I was very interested in chemistry; I was interested in physics. And what Paul did is he showed how structure pulls chemistry into biology. So instead of biology being this magical thing that we cannot understand, you could actually, once you understood the structures of the right macromolecules, define in chemical detail and explain how biology works. And I just thought that was unbelievable. I decided to do my graduate work in structural biology, and I was trying to figure out what to do. At the time, Wayne Hendrickson had just come up with amazing MAD (multiwavelength anomalous diffraction) technologies that allowed us to use multiple wavelengths to solve structures.

Wayne asked if I would like to work on the HIV receptor, CD4, which Richard Axel had just cloned at Columbia [5, 6]. It was sort of a whirlwind of things.

And in structural biology, one thing that was amazing was that, as just a graduate student, you could have your pick of whatever projects there were, because structures were difficult; they took years. And so, you could find what you wanted to do. Wayne gave me CD4, and it was actually a race [7, 8]. But then things like gp120 took a decade, and there weren’t that many other people racing to get that one, though many people tried.

## NSG

Yeah, that was a tough one.

It speaks really highly of you that you were able to bring that to fruition.

## PDK

Thanks. So, Paul and Wayne were there. I do want to mention one other person, though, my sister Ann D. Kwong, who’s 7 years older than me and a virologist. She partly guided me through this. When I was an undergraduate at the University of Chicago and looking for different work/study positions, she told me there was a position in Paul Sigler’s lab. She had spent a summer building a tRNA molecule in his lab; she went into virology and got her PhD, so she started me off. Once I hit Paul’s lab and saw what one could do with structure, I was like, oh my gosh, this is amazing.

## NSG

That’s great. I did not know about the sibling-directed direction. So, what have been the chief interests that drive your research activities? Is it fair to say that it’s using structural biology as an entry point to studying mechanism in biology at a molecular level?

## PDK

Yes, absolutely.

Often, we understand organic chemistry. And so, we can reduce these wonderful biological molecules from the macromolecular straight to their chemistry, and the way to do that is, of course, with structural biology, because once you determine the atomic-level structure of a macromolecule, it’s just complex organic chemistry, which we can understand. And if you can understand something, you can figure out its mechanism, etc. The ability to manipulate things and do things with them is really exciting. While the concept existed, the 1990s were when structure-based design became effective. That occurred, in part, with HIV. The development of things like RT (reverse transcriptase) and protease inhibitors really started structure-based design because they showed that if you had the right structures, you could come up with all sorts of ways to manipulate and impact biology. So that was exciting to see.

## NSG

So, when you say RT, you mean reverse transcriptase

## PDK

Yes, I’m sorry, reverse transcriptase inhibitors or protease inhibitors as well as basically different HIV inhibitors that were designed based on atomic-level information or by structure-based drug design.

## NSG

So, structure-based drug design was sort of a clue to you that structural biology in an immunological context could be a productive path forward.

## PDK

Well, I was more interested in what you could do with structure and the ability to make drugs. You had these fascinating mechanisms. And, at the time, any structure that came out was on the cover of a glossy magazine, so you knew that they were impactful. The ability to make drugs that could do more than contribute to understanding was really something. And when I got into looking at viral proteins like the HIV envelope glycoprotein, gp120, the structure that I solved in 1998 could have gone in many different directions [1].

One direction was to take it into drug design. There are small molecules that bind to gp120; you can look at the structures, etc. Or vaccine design, because it’s the only target of neutralizing antibodies. When I was trying to figure out what to do, the reason why I chose vaccine design, in part, is because at the time there were other targets of HIV where you could use drugs with fairly conserved sites — on protease, RT, or integrase — which would make much better drug targets, whereas there is only one target for a vaccine, and that’s the envelope glycoprotein.

And so since I had something that was unique, I think it was Gary Nabel who basically said, look, there are a dozen HIV inhibitors, and there will be more; there is no vaccine, so if you want to work on something new and impactful, try vaccines. What Gary Nabel didn’t say is that it’s also very difficult. That’s partly what drove my interest. Not only was it fascinating science, but you could actually do something useful for human health and make an impact.

## NSG

Your answer prompts an additional question, which is: Are there any small-molecule inhibitors focused on gp120 at this point?

## PDK

There’s nothing licensed, but we did try to develop several compounds. It turns out, for example, that at the position where CD4 binds to gp120, there’s something called the Phe43 pocket. There are inhibitors that sit directly in that pocket, and there are a bunch of small molecules that can bind there and inhibit HIV. So, there are a number of different compounds that are pretty good. But it’s just that the molecules don’t work as well as the inhibitors against the super-conserved intracellular enzymes. You also have the diversity of the envelope that confounds small molecules just like it confounds the immune system.

## NSG

That’s interesting, and so they have made progress. It’s just that they’re not at a practical, clinical-use level at this point. And, compared to these alternative targets, it’s not as good.

## PDK

I think that’s correct, Neil. If you were to design different things, there are a number of leads that target the HIV envelope, but none have made it to FDA approval.

## NSG

You mentioned in earlier discussions or correspondence with me that there are really multiple kinds of structure-informed, let’s say, design, that help with creating vaccines in ways that go beyond the traditional ideas of just plucking out a particular antigen and using it as a subunit vaccine, or killing a virus and using it as a dead vaccine or live attenuated virus.

And you identified three different types of structure-informed design. One is structure-based design, which I think RSV, respiratory syncytial virus, is a good example for illustrating. And then antibody-based design and epitope-based design. Could you just expand a little bit on each of those?

## PDK

I gave a talk, I think in 1999, that Norm Letvin saw at the NIH, and I think he told Gary Nabel, who was the person who set up the Vaccine Research Center, (Norm Letvin was one of the initial advisors) that he thought what I proposed for structure-based vaccines was an interesting idea. The issue that’s different about structure-based drug design from vaccine design is that in structure-based drug design you could take the lead compound, crystallize it in a complex with the target, and visualize the direct interactions — straight organic chemistry — of how the drug was impacting its target and therefore manipulate and change it directly. That’s structure-based drug design.

The problem is that for the HIV vaccine, you’re trying to manipulate the immune system. And you could crystallize antibodies, of course, the products of the immune system, with your macromolecule, your antigen or your immunogen, but it wasn’t possible to crystallize the immune system with the immunogen. We had to come up with some way to do it—a lead or a substitute for it.

The initial ideas that had to do with structure-based vaccine design and structure-based drug design go back to the 80s and 90s. I think Godley and Wiley did an interesting experiment with influenza hemagglutinin (HA), where they put disulfides that fixed it in the prefusion conformation [9]. Not a better vaccine at all. And that’s partly because a lot of these early efforts in the 80s and 90s focused on just the viral part. The thought was if we understand the virus, manipulate the envelope, and get it just right, we’re going to get a good vaccine, and they completely ignored the immune system, which is the actual agent that you want to impact.

And you mentioned structure-based design, which is to take the molecules and manipulate them and change them, which is what we did. In 2010, I think, we got the initial structure-based design of the HIV envelope. We took the gp120 alternate structure and stabilized it in such a way that it would present a CD4-induced epitope. CD4-induced antibodies are very, very broad. We got this to work. You don’t get them normally by immunization with gp120. But once you stabilized gp120 — specifically the bridging sheet—we did elicit CD4-induced antibodies. It’s work we did with Rich Wyatt and Gary Nabel [10]. The problem is that the CD4-induced antibodies that we elicited at that time, even though they’re very broad in their neutralizing activity, don’t neutralize primary isolates, only laboratory-adapted ones.

And so, we learned that you have to consider the right antibody. If you don’t have the right antibody to guide development, you can get a vaccine that elicits the wrong type of antibody, and it’s not useful. After that initial attempt, we decided if we’re going to develop a vaccine, we needed to think carefully about the antibodies we would like to elicit. This led, in part, to our work on RSV with Barney Graham. At the time, we knew that there were prefusion and post-fusion conformations, but we chose an antibody called D25, which has super-potent neutralization. We didn’t know formally all the structures at the time when we were investigating this. Jason McLellan, who was a postdoc in my lab at the time, found D25 while searching the patent literature, and we thought the conformation recognized by D25 must be something useful.

It took us about a year or two to get the structure. Immediately after we got the structure, which is just D25 binding to a soluble form of RSV, we could then see exactly the conformation that was susceptible to D25. And based on that structure, we could then fix its conformation [11, 12].

That’s also part of this antibody-guided design, because without D25, we wouldn’t have had a way to capture RSV in its antibody-susceptible conformation, and we would not have known how to fix it. There weren’t hundreds of antibodies, there was just a super potent one that stood out. That became the basis for the first antibody-guided design, which led us to design DS-Cav1. We basically took immunogens and we were looking for recognition of an antibody, and we could stabilize it by enhancing the stability or the enhanced recognition of that antibody, in this case, it’s D25.

And then we ended up taking many of the same procedures with other antibody-guided vaccines, where we had something super potent or something we really wanted to target, and that was used to guide the development.

Initially that was just binding, as I’ve described, but ultimately we realized effective HIV antibodies, unlike RSV ones, were very difficult to make and that oftentimes they had long, unusual maturation histories, or they had unusual recombinations (editor note: ie, the resulting variable domain modules [VH + VL] of successful antibody gene rearrangements) that gave rise to them.

So, the B cell ontogeny that produces these antibodies, we realized, was something we had to manipulate to get more effective HIV antibodies. And so antibody-guided vaccine design ultimately transitioned into B-cell ontogeny-guided vaccine design [13, 14]. And it’s finally succeeding, actually, which is sort of incredible. In the next couple of months, you’re going to see a number of papers coming out in *Nature*, for example, where they finally achieved high enough titers with broadly neutralizing antibodies (bNAbs) [15–17].

I was able to use antibody-guided design. In this case, we chose the right target, which is the fusion peptide-directed epitope. We took the fusion peptide-directed antibodies and figured out how to elicit them by vaccination. We got antibodies with high breadth that would cover at least 50% of HIV-1 diversity. That was in 2016 with John Mascola that we found the right epitope [18, 19]. In 2019 we got bNAbs in rhesus macaques [20].

Then we realized that in order to protect, you can’t just generate antibodies, monoclonal antibodies. You have to achieve high enough titers in sera so that when one encounters a pathogen, the antibodies can neutralize it. While we could get the bNAbs at the B-cell level, we have not succeeded with fusion peptide in getting the titers high enough to fully protect by vaccination alone.

At the time, we were outliers; we were the only ones with vaccine-elicited bNAbs, so other groups had not yet had these problems. They were still trying to initiate the antibody lineages and get them started. In the last 4 or 5 years, while we were trying to get higher titers of fusion peptide, those other groups caught up and some have now passed us.

There is a wonderful paper by Javier Guenaga and Rich Wyatt in *Nature*, in which they’ve used heterologous boosting and multivalent Env immunogens displayed on liposomes, which will be a promising way to go forward [15]. I heard Rich speak about this at a recent Keystone meeting. And then we heard a second talk by Jon Steichen, who’s at Scripps, working with the BG18 epitope in V3. They used sequential heterologous boosting, including multivalent immunogens, to achieve high antibody titers as well [17]. So, I’m really excited because now the field is moving in this direction.

One of the papers I’ve been working on with George Shaw is part of this collection of papers in *Nature*. In our case, we focused on V2 apex antibodies [16]. We optimized the priming and then figured out how to boost based upon simian/human immunodeficiency virus (SHIV) experiments.

I’m probably giving you a lot to unpack, but basically, we started with structure-based design only. Then, we realized we had to layer antibodies on top of that. We had to identify and to understand the antibodies we wanted to elicit. Once we did antibody-guided design, it eventually evolved into understanding the ontogeny of the antibody, how to prime it, how to enhance priming, and how to shepherd the antibody response to broad neutralization at the high/potent titers that would be impactful in the vaccine setting.

## NSG

I had been familiar with the group at Scripps and the work they did, showing that if they were able to induce an antibody, a B-cell clone that represented the root of a good lineage with respect to bNAbs at the end, that they could lead the evolution of that clone with different immunogens over time.

Are you able to now get bNAbs of high breadth, meaning they can react with many different strains of HIV, through just using a single immunogen in multivalent form? Is that what you’re saying?

## PDK

Right. So, we do require multiple steps. Hopefully we can reduce it to at least three. But right now, it’s multiple steps. In order to figure this out, we had to actually start doing the sequencing of donors that had these antibodies. So Tongqing Zhou did the B12 structure from Dennis Burton that was pretty broad, 30% broad; it was on the cover of *Nature* in 2007 [21]. Then the field started finding more of these that were really broad at the 90% level, and ours was VRC01, which we found in 2010. Tongqing and I worked with Gary Nabel and Bill Schief to create probes, and Gary Nabel and John Mascola used those to sort, and they found VRC01 [22].

So Tongqing and I did the structures [23], and then we moved forward with sequencing with John Mascola and Larry Shapiro. It turns out that if you look for VRC01-like antibodies, when you sequenced the B-cell repertoire, you could watch them develop, these antibodies change by over 50% in their sequence in order to evolve from the initial recombination to the mature bNAbs with high breadth [24].

## NSG

When you say 50%, are you talking about the whole V module VL and VH or just VH?

## PDK

So the variable domains of the bNAbs of VRC01-class antibodies can vary by about 50% in their heavy- and light-chain sequences at the amino-acid level. The difference is about 30% at the nucleotide level. That means that VRC01-class antibodies from different donors, even though they’re the same class and recognize HIV the same way, can have enormous differences. That’s really interesting, but it makes vaccine design very hard.

In order for us to produce that response with a vaccine, you would have to initiate the B-cell lineage and boost it, which is a process that takes a decade or so in humans. We thought this was going to be really difficult. This is why we thought you have to choose the right antibody to target.

Bill Schief and others at Scripps took on the challenge. They developed knock-in mice, and they succeeded in priming. But the problem with antibodies like this is you have to get to 50% evolution to get them to finally mature. That’s going to be very difficult. And even though they could get things further in knock-in mice, they could never get anything in humans or in monkeys. Monkeys don’t have the right V genes, so it was difficult for them to work it out.

## NSG

And again, to clarify for listeners, when you say 50%, you’re talking about the entire sequence of VH and VL, not just the hypervariable regions.

## PDK

An antibody heavy chain has four domains, and the light chain has two domains. The N-terminal two domains of the heavy and light chains, the VH and VL, together have about 250 amino acid residues that undergo somatic hypermutation. In the VRC01-class antibodies, about 50% of the amino acid sequences are changed. You get about 100 residue changes.

## NSG

Does that include positions in the framework regions?

## PDK

Absolutely. All over.

## NSG

That is interesting because people have been taught for years that there are hypervariable regions and there are framework regions, and only the hypervariable regions are involved in binding, which we’ve known for decades isn’t entirely true, but in these cases, do you know from structural analysis that some of those changed framework residues are actually making contact with antigen and or influencing contact by hypervariable residues?

## PDK

It’s much more the latter that you get influences of other regions to appropriately present in different ways. It’s more the latter that the framework regions help the CDRs in terms of their recognition.

## NSG

One reason I ask is that people often interchangeably use hypervariable region and complementarity-determining region. And I think there’s a difference there. I’m interested in your response to this proposal that hypervariable residues can only be determined by sequencing a population of antibodies. Complementarity-determining residues can mean residues that have a statistically high probability of being contact residues in one antibody-antigen complex or another versus in any specific complex actually making contact, and then it also raises the question of what counts as a contact.

But in that case, there are two different definitions that are possible. One is population-based, and one is a specific antibody with a specific antigen.

## PDK

I think both definitions are helpful. When you look at, for example, donor 45, we studied him over the course of 15 years, and we saw enormous differences. Things changed in CDR H3 length, for example. You could get some of the shorter ones of 12 residues in length like with antibody VRC01, or you could go to something like antibody VRC08 [25]; it’s an antibody from the same lineage (eg, sharing a common origin from a single germline B cell, whose clonal types evolved for more than a decade), and it’s over 20 residues in length. So, there are insertions, deletions, and all of these changes throughout. This lineage evolved a lot. And what we realized is that it might be difficult to get more – high levels of that antibody with that degree of evolution – with a vaccine.

It wasn’t until 2014 that we saw another type of antibody. We were analyzing a donor from South Africa, CAP256, with Lynn Morris and Penny Moore, and the antibody that was broadly neutralizing in this donor bound at the V2 apex at the top of the viral spike [26]. We saw much less somatic hypermutation with this antibody. I think the hypermutation frequency was 10% or something, much lower, and with good neutralization coming up almost right away.

And so we thought the V2 apex of the HIV-1 Env trimer might be a lot better target surface for a vaccine. We saw that in 2014. The problem was that we didn’t know how to make really good envelope trimers in 2014 that bound well to the V2 apex bNAbs, other than just a few strains, such as strain BG505, with which we solved the trimer structure [27] and figured out how to stabilize [28] in the prefusion-closed state. So, when we started asking ourselves what the best immunogens were to elicit these V2 apex antibodies, we wanted something that would interact with their germline precursors, to start the lineage off.

We took the V2 apex bNAbs, there were about 5 of them that had been identified at that time, and we reverted them to their germline versions, to what we thought were the initial recombinations. Then we asked whether there were any HIV strains that were sensitive to neutralization by these germline-reverted antibodies. Because neutralization tells you that you’re getting an interaction.

And lo and behold, we did find a few strains [29]. There were about 10 or so that were sensitive to neutralization with the V2 apex bNAb germline precursors. We could then take these special strains and hopefully use them to immunize and induce more of the V2-apex-directed antibodies. It turned out that was difficult because we couldn’t make the V2 special strains as well-behaved trimers. All of the trimers we made failed to bind well to the target V2 apex-germline precursor antibodies. And while we did ultimately succeeded in making well-behaved trimers of virtually any strain [30], in 2015 we also tried a different approach.

There are a couple of different ways that you can make envelope, as a soluble trimer or on an infecting virus. We decided to work with George Shaw on the second: to make an SIV that replicated the human immune response to HIV in a monkey. That is simian immunodeficiency virus (SIV), the virus that we’re manipulating, but changing the envelope so that the entire outside is HIV and the entire inside is SIV. You can infect monkeys, but you get HIV envelope responses. That technology came in 2016/2017. It turns out that you needed a very specific mutation, a tryptophan at position 375 or a big hydrophobic residue to do that. And then you could take HIV envelopes and, if you infected monkeys, obtain HIV-1 responses in sera, including bNAbs [31]. So that was 2016 that the *PNAS* paper came out [32].

Then I said to George, let’s try these special strains that we can never make as trimeric immunogens and see what actually happens with SHIV infection. And there were about 10 of the special strains. The best were ZM233, Q23.17, and some others. It took like 10 years to do this; there are recent papers just published this year where Rumi Habib (a student in George Shaw’s group) took over 100 monkeys and showed that SHIV’s infected with special strains, had a much, much higher, about 50% chance of eliciting a bNAb response. This showed it was working in part [33].

And then, working with some of the initial data that we had, a new approach that Kshitij Wagh put together created what’s called CAP256.OPT4, in which one uses neutralization-specific signatures to understand what was creating those special strains, why they were good at engaging V2-Apex bNAb germline precursors, and how to create a much better immunogen [34]. And so that’s a paper in *Nature* from George Shaw’s group [16]. Lori Marchitto is the first author on it. That work creates this V2 apex immunogen. It’s about a 30–fold increase in priming compared with even the Q23.17 special strain, which already works okay for induction of V2 apex bNAbs, but this is even 30-fold better [15, 33, 35].

What’s even more remarkable about this paper is what happens with SHIV infection. If you start with those modifications, you get bNAbs within 6 months. But then we could actually figure out by looking at the coevolution of the virus what changes the virus had to make to teach the immune system to get not only just a few new titers, but to get really broad bNAbs, including strains with glycan at position 130 and other strains that other people haven’t gotten.

By doing this experiment, you could show with SHIV that it did work. You get broadly neutralizing antibodies, but then we could understand the pathway through which those developed and then work backwards and immunize. And they’ve done so, and the Marchitto paper shows high-titer responses coming from this immunization approach, using what we learned from SHIV as clues to guide the development of the immunogens.

This is a lot to understand: how structure-based design starts using clues from many different things, including from SHIV coevolution to trace back and figure out what the pathway was that the coevolution between the virus and the immune system led to bNAbs. That you could recreate, to get the right immunogens to develop broadly neutralizing antibody activity [16, 31, 33, 36, 37].

## NSG

So, does that mean that you would now have a better idea of what serial immunogens would lead you on a path to the bNAbs that will be able to neutralize a very broad array of actually circulating HIV strains?

## PDK

Yes. Neil, absolutely. For very specific targets. For fusion peptide, we could vaccine-elicit bNAbs that were 50% breadth. That goes back to 2019 [18, 20]. We had not succeeded in getting the titer high enough from vaccination alone — only after SHIV boosting [38]— but we’re trying now, because we figured out that there are these multivalent immunogens that may allow us to do so. We’re creating those now. And I think those will go into animals with Dan Barouch in about 3 months.

And so, we are at high titer already for the V2 apex; those papers are coming out. There are papers that are already coming in that context. And I think for other epitopes like V3, the results from Jon Steichen and Bill Schief are also quite exciting [17].

So even though for HIV we have drugs that are amazing-for example, 1 or 2 injections of lenacapavir over a year will protect you from HIV prophylactically- we are finally getting to high titers that might have an impact with vaccination.

Is it too little, too late? It’s hard to say. One thing that I’m super excited about is that, with HIV, you have all the tools to figure out how to do vaccine development. I believe that these will be useful against other vaccine-difficult pathogens where you need a particular CDR H3 or where you only have a couple of bNAbs that work—how do you get those vaccines? I think the technology coming from HIV could impact all these other areas as well.

## NSG

Yeah, I mean, you know, influenza, hepatitis C, Covid or other coronaviruses.

## PDK

That’s right. What this means is that if you can really use this antibody-guided vaccine strategy to develop vaccines, anytime you have a good, broadly neutralizing antibody that’s really effective on its own, you should be able to recreate the antibody’s neutralization with a vaccine.

And if you have pathogens that are not touched by antibodies, I think like *Staphylococcus* (staph) infections, you get all these antibodies against staph and they don’t do anything. But if you have pathogens that are neutralized by an antibody through its binding capability, you should be able to create vaccines with the antibody-guided structure-based vaccine technology.

## NSG

So, here’s a point that has been of interest to me for many years.

Some people equate higher affinity antibodies with greater specificity. And I have actually questioned that. I don’t know if you ever saw a book by Irun Cohen [39], who is an Israeli immunologist, called “Tending Adam’s Garden?” In that book, he defined a relationship between affinity and specificity. I’m not remembering which way he defined it. And then Jonathan Howard reviewed the book in *Science*, and he had a very favorable review, but he had the exact opposite interpretation of the relationship between affinity and specificity [40].

So I wrote a paper suggesting that there is no simple relationship. It depends on the biophysical source of the high affinity [41]. Because if it’s hydrophobic, higher affinity may indicate less specificity. But if it’s hydrogen bonds and complementarity of a structural nature, then it might be that they correlate closely. I didn’t actually use HIV as an example in this paper, but in retrospect, it would have been potentially valuable because some of the best broadly neutralizing monoclonals are not only much more broadly reactive and therefore less specific in an antigen sense, they also bind with higher affinity than many of the antibodies that are not broadly neutralizing. But you can also distinguish between specificity on an antigen basis and specificity on an epitope basis. And those can be at odds. You can be less specific for the antigen in total, but more specific for a particular epitope on that antigen, which is approximately shared with other different antigens as opposed to a polyclonal collection of antibodies, it would be recognizing many epitopes at the same time on average.

And so, you know, these are complex concepts, but they’re treated in most teaching as if they’re absolutely clear-cut, and they’re not, in my opinion. So I’m curious, would you give any merit to those arguments?

## PDK

I do agree with you that the relationship between affinity and specificity is difficult. And the reason why it’s difficult, for example, is that say you have an antibody with very, very high affinity to a sugar molecule, right. But that sugar molecule might appear on 40 different glycosylated viral proteins. And so it’s going to bind tightly to every single one. And if you increase the affinity, it is still going to bind tightly to every single one. And it’s specific for that sugar, sure, but it binds tightly to many other underlying targets. That’s just the example where you can get something with high affinity, great specificity for that sugar, but then in terms of viral pathogens, it just binds to every single one that’s glycosylated. So, you can display a lot of things on the cell surface, etc. And there are other ways. That’s just one example. But that’s why I agree with you that the linkage between these big concepts can be quite complicated. I can see the counterargument that, if you have super high affinity, you could have increased breadth, in part because something that would otherwise bind relatively weakly might still bind sufficiently to neutralize because the antibody has high intrinsic affinity, which sort of overcomes the lower relative affinity. There are many ways you can come up with these in special cases versus not, but I bet you can come up with counterexamples in each case.

## NSG

So my point was really just that it can be complex, and there is no single generalization that will work 100% of the time. And essentially what you’ve just described is an example of what I would call high specificity for an epitope, that then gives you lower specificity for the different antigens. It’s not selective at the antigen level but is at the epitope level.

Anyway, I think we can move on. I think what hopefully people will take away is that what you’ve managed to accomplish with your colleagues and with other labs in the game is to work out just an incredibly challenging vaccine scenario where because of the high diversity of the virus, it emphasizes how lucky the early vaccine developers were, where you could just kill a bacterium or a virus—if that word works for viruses — inactivate them and stick them in, and you’d get essentially something that worked as an immunogen. But with these highly variable pathogens and viruses in particular, it takes just an enormous amount of effort to find the right way to induce an effective immune response.

## PDK

I totally agree with you Neil. It was Maurice Hilleman who said isolate, inactivate, and inject. That works for many, many different viruses, certainly, and many different vaccines have come from that. But the problem is if you come up with a vaccine-difficult pathogen like HIV, influenza — all the sequence-diverse pathogens are somewhat difficult to get vaccines that way — what do you do? And what I’m hoping to do is say, well, if you can find an antibody that can neutralize that pathogen, then here’s a way that you might be able to create similar antibodies over and over again by vaccination.

The other thing that you can do, of course, is you can use antibodies by themselves. And so antibodies are in therapies that are moving forward. And so if I look at the three big areas that I’ve made impact, it’s vaccines, antibodies, and structure, like viral structure — mainly HIV is where I’m mostly cited — but Larry and I along with Ryan Roark just determined the prefusion structure of HSV-1 gB (herpes simplex virus 1 glycoprotein B, the fusion machine of herpes virus responsible for viral entry) [42], and I thought that was really fascinating as well and very, very different because that viral envelope escapes almost all antibodies that bind to it. Basically, you have prefusion gB-binding antibodies, but even though they bound a prefusion gB, they also bound postfusion. And most of the antibodies that bound prefusion gB don’t neutralize herpes entry at all.

And so that’s something that’s really interesting as well, because it turns out that the virus envelope appears to change shape and not be impacted by the fact that an antibody might bind to it. So yeah, very, very different mechanisms of neutralization. And that’s why once you find an antibody that effectively neutralizes, that’s what you go after.

## NSG

Are there any neutralizing antibodies for that antigen?

## PDK

So gB, which is the entry machine for herpes, for a long period of time people were looking for prefusion-specific antibodies, and they had some that were sort of not that interesting because they would bind to areas like the fusion loops, and — while that’s something of interest — they weren’t fixing conformation, just blocking something. Or there was, I think, one domain that you could get some binding that appeared to be prefusion specific.

It wasn’t until we stabilized the structure of gB into its prefusion conformation and then immunized that we found antibodies that were prefusion-specific — antibodies that in themselves stabilized the prefusion structure. And it turns out that there are basically several ways that you can neutralize gB, because if you looked at the structure of the prefusion gB from herpes, it was made up of all these domains, and so the prefusion and the post-fusion basically have the same domain structure and gB just rearranges a bit.

## NSG

So the individual domains remain sort of conserved structurally, but their quaternary relationships change?

## PDK

Yes. Think about your arm and parts of your body. They’re all conserved in structure. They just move relative to each other and you end up with a different body shape, you know, this way (motions with arms) versus that way.

## NSG

So this is sort of escape at the molecular dynamics level as opposed to the mutational level. Is that accurate?

## PDK

I would say it’s at a structural level: that there’s a structural mechanism that allows gB to refold. And antibody generally doesn’t prevent gB refolding into other conformations. And so one of the reasons why I think this is really fascinating is that it turns out that there are still specific ways to fix conformation, such as if you have antibodies that bind two domains, cross-bind like quaternary antibodies, they neutralize, they fix things as well.

And so you can either bind across domains or you can bind to certain regions that are called refolding regions that actually do refold. And so we had one antibody that bound to a refolding region, and collaborators actually found other antibodies that bound to two different domains simultaneously [42, 43]. And they also neutralized. So there are very specific ways to fix gB conformation that fit with our molecular understanding of how domains move. And if you fix them, you can get very potent neutralization.

## NSG

I think that’s a really extraordinary example where the standard way we think about, at least my experience is the way we teach students is that you make an antibody and the virus escapes that immunity by making a mutation, but you’re suggesting there are non-mutational mechanisms that allow escape.

## PDK

Oh yes. Viruses have all sorts of different ways to escape things. For example, antibodies that recognize the CD4-binding site of HIV gp120 are very common. There are all sorts of ways to do that. But the HIV viral envelope has conformational masking [44], which holds the envelope in a threefold prefusion-closed state that only multimeric CD4 can trigger to allow things like co-receptor to bind. And if you just get antibodies that bind to each CD4-binding site separately, they don’t do the full-on triggering. And so it’s very difficult to get multimeric molecules that can trigger. So yes, there are all sorts of structural ways that viruses use to protect their envelopes — folding constraints or things to escape the immune system — other than sequence variability.

## NSG

We discussed a little bit about this concept of glycan shielding, and can you just briefly comment on how that works?

## PDK

Yeah, glycans or sugars are really fascinating. Glycan shielding is because the body makes N-linked glycans that it attaches to proteins. But it turns out that if you make a standard N-linked glycan or sugar, the sugar is about the size of an antibody’s recognition site. And the sugars that are placed on the virus by the body’s own machinery and the sugars that are placed on human cells are the same.

And if you have an antibody that binds to one of these sugars, oftentimes it binds to the other — including self glycans on cells. So in general you can get antibodies that bind glycan, but if they bind to them tightly they’re going to be considered self and they’re going to get removed from the B-cell repertoire. And so it turns out that if you’ve got lots of glycans on the surface of the HIV-1 envelope, it creates an entropically difficult pathway for the antibodies to bind to the protein underneath that glycan shield [45, 46].

And if you get recognition of the glycan instead, then you get recognition of self and the antibodies are lost. So having glycans cover much of the HIV envelope surface enables a very efficient way to have large parts of the surface shielded from the immune system because of all the glycan.

## NSG

That’s very interesting. Do you foresee in the future that we might be able to predict binding and neutralization using machine learning/artificial intelligence methods, in other words, just by knowing structures? So if you used AlphaFold or the latest version of AlphaFold to predict an antigen structure, and you had an antibody and you wanted to know is it going to neutralize, are we close to that, or do you think that’s possible? Any thoughts on that?

## PDK

That’s an interesting question. Where are we right now with predicting neutralization from sequence? We can fold a lot of structures for antibodies. You can calculate what the structure could be, and those AlphaFolds have become pretty good at doing this. From the viral proteins we can understand what the virus looks like. But you take an antibody and to say where the antibody binds on a pathogen can be very difficult. That’s the first part. Partly because, if you have induced fit, any structural change to both sides, the model has to figure out how each part changes and where an antibody will bind.

So that’s the first problem, and certainly that’s a big issue at the CASP (Critical Assessment of Structure Prediction) competition right now, where they’re trying to figure out what can be done. Finding how biomolecular complexes interact is a big challenge. My guess is they’re going to figure that out and they’re going to overcome this challenge. If you can figure out really good antibodies in that way, then I think we can start doing a lot more in silico. But that’s a big challenge right now, which is why experimental structure is still useful. Because if you want antibody complexes, they can’t calculate where antibodies bind so well. So that’s very, very difficult.

## NSG

So just to clarify for listeners, CASP is a competition that for years was devoted to predicting three-dimensional structures from sequences, amino acid sequences of proteins. And now, has it progressed to focusing more on interactions between different proteins, or between proteins and other ligands?

## PDK

I think CASP always had different levels. There are still some predictions of individual structures, but CASP has always included interactions. And such interaction prediction was abysmally bad before, and now they’re getting better. So, I’m sure that someone’s going to come up with technology to figure out how to do it.

## NSG

Yeah, that’s worth noting. What are your thoughts having been both an investigator at the Vaccine Research Center and now being an extramural investigator? In other words, looking back, can you make a comparison between what it was like being an investigator at the VRC in, for example, 2018 versus being an investigator at a university outside the NIH in 2018?

## PDK

I think the main issue, in terms of the way research funding is structured, because we all are dependent upon funding for research, is that in the intramural community, you get evaluated every four years. What happens is you do the work, and then you write it up, and you say, “This is what I did the last four years.” Then they decide whether your funding increases, decreases, or you’re fired, whatever they want.

## NSG

But it’s largely based on past accomplishment as opposed to future plans.

## PDK

Right. It’s largely all past, and in the extramural community, when you apply for a grant, it’s all about what you propose to do. So those are the big differences. Of course, nowadays when you apply for a grant, you better already have completed all your aims and the preliminary science data so that you can get the grant, because otherwise they may say you can’t do what you propose. So, most people have already finished some of the work anyway.

But that’s the big thing I noticed that, in the intramural community, they trust you enough that you’re given the money ahead of time. Afterwards, the question is do they keep supporting you or not? And that big change is important for a number of reasons.

The first is that if you’re planning something, you’re given money and you’re busy working away. You collaborate, you do all those things, but you’re not forced to think ahead about what you plan to do and pull together the collaborators.

That’s part of the process when you’re on the outside and writing grants. You really think about how you’re going to do this, what it means, and who are the right people, etc. There’s a bit more thinking when you’re setting things up and planning what you’re going to do, because in the intramural community, the money is there; you just start doing it. The barriers are much lower to get things started on the inside.

Oftentimes on the outside, if you really want to get something started, you just start anywhere, with whatever money you have available. In practice, I think it’s not that different. But on paper, one system funds you before the work and the other, after. I think that makes a very significant difference.

The other thing is that funding from inventions actually happens differently. If you’re intramural, there’s a cap on how much you can receive for your patents. Like universities, the NIH gives inventors about 25% of the patent royalties, but unlike most universities, they max you out at a certain amount.

I think at the NIH now, the maximum is $150,000 per year, which is pretty decent. And we knew that going in. For the RSV royalties, we knew about how much it would generate. I’m very proud to say that we maxed out about 40 inventors at the NIH from the RSV patents. So, there’s a bit of funding coming in, which is very helpful because it really drove a lot of things. In the lab, we called it the RSV pension fund; so anyway, that was somewhat lucrative. But, on the outside it’s very different. You can make quite a bit more. You’re not maxed out at all.

## NSG

So, in closing, do you have any thoughts you are willing to share on how graduate education can be improved? Did you have graduate students when you were at the VRC?

## PDK

They do have students at the NIH in a number of different ways, with programs that basically embed people. I had one graduate student, Leo Kong, who spent two years with me, and then he spent two years at Oxford with Quentin Sattentau. I think Leo did the two years with the NIH first, and then he got the degree from Oxford. So, there are a bunch of programs that let you have students. But most of the people I had at the NIH were postdocs.

## NSG

Are you taking graduate students now?

## PDK

Yes. I have one that just finished a rotation with me and another one that is working with us right now.

One of the reasons I wanted to come back to Columbia University is to set up a lab with my friend Larry Shapiro, who has done all the Antibodyomics work with me. We have a joint lab, and in that, there are two potential students that we’re working with right now. But students or postdocs, they’re all students; it’s just different levels of training.

For trainees, I think it’s really important they find something they’re really passionate about. When I was in Wayne Hendrickson’s lab, and we were doing structures, it was crazy that you could just pick a project that might be covered in the *New York Times*. It was something really exciting.

There are so many things you can do with science these days that if you’re making a choice, do something exciting, something you’re super excited about, and hopefully it’ll be impactful for human health as well. So that’s the only thing that I would say: find something that’s going to make an impact and something that you’re really excited about.

## NSG

And for young people who are considering careers in biomedical research but haven’t really made the plunge yet, do you have any guidance to offer?

## PDK

One of the things that I think is great about biomedical research is that there are so many different ways that you can approach this. When I was initially interested in physics, for example, a lot of these experiments with physics were like these big thousand-person experiments to find some unknown particle, and it would take a decade or something to do that. And you can have enormous teams.

Not to say that’s not true in biomedical sciences, because there can be enormous teams with projects like sequencing the human genome, but a lot of biomedical research is just a couple people, and so you can really have a big impact there. And what I would suggest is to try your hand at research, see if you like it. It’s for some people, and not for others. Some people like the uncertainty of finding something new. Other people hate the uncertainty even if they’re finding something new. If you find that the idea of new frontiers is really pushing you, certainly try it.

I think that’s really helpful because, say, you want to go into business and get your MBA; you have no idea what it’s like until you start doing the work. Whereas one thing that’s actually nice about graduate school is you can figure out what it’s like to be in a lab. You can figure out what the research is like before you fully go into it.

Another example is a junior in medical school: you’re taking all these classes, but that is not really what it’s like to be a doctor. Your first two years, you’re like, “Oh, this is great stuff.” But then your third or fourth year, you find you faint at the sight of blood or whatever.

So, try your hand at it. If you’re taken and you think, “I want to do more of this.” Great. If not, well, there are many other ways to contribute. Not everything has to be with research.

## NSG

Well, this has been very informative, Peter, thank you so much. It’s truly been a pleasure to speak with you.

## PDK

Thank you, Neil. It’s been fun on my part, too.

## SUPPLEMENTARY VIDEO

Supplementary videos are available at the *Pathogens and Immunity* YouTube channel.
